# Magnetic resonance imaging based deep-learning model: a rapid, high-performance, automated tool for testicular volume measurements

**DOI:** 10.3389/fmed.2023.1277535

**Published:** 2023-09-19

**Authors:** Kailun Sun, Chanyuan Fan, Zhaoyan Feng, Xiangde Min, Yu Wang, Ziyan Sun, Yan Li, Wei Cai, Xi Yin, Peipei Zhang, Qiuyu Liu, Liming Xia

**Affiliations:** ^1^Department of Urology, Union Hospital, Tongji Medical College, Huazhong University of Science and Technology, Wuhan, China; ^2^Department of Radiology, Tongji Hospital, Tongji Medical College, Huazhong University of Science and Technology, Wuhan, Hubei, China; ^3^Department of Research and Development, Infervision Medical Technology Co., Ltd., Beijing, China; ^4^Department of CT & MRI, The First Affiliated Hospital, College of Medicine, Shihezi University, Shihezi, China

**Keywords:** testicular volume, magnetic resonance imaging, deep-learning, T2-weighted imaging, ResUNet

## Abstract

**Background:**

Testicular volume (TV) is an essential parameter for monitoring testicular functions and pathologies. Nevertheless, current measurement tools, including orchidometers and ultrasonography, encounter challenges in obtaining accurate and personalized TV measurements.

**Purpose:**

Based on magnetic resonance imaging (MRI), this study aimed to establish a deep learning model and evaluate its efficacy in segmenting the testes and measuring TV.

**Materials and methods:**

The study cohort consisted of retrospectively collected patient data (*N* = 200) and a prospectively collected dataset comprising 10 healthy volunteers. The retrospective dataset was divided into training and independent validation sets, with an 8:2 random distribution. Each of the 10 healthy volunteers underwent 5 scans (forming the testing dataset) to evaluate the measurement reproducibility. A ResUNet algorithm was applied to segment the testes. Volume of each testis was calculated by multiplying the voxel volume by the number of voxels. Manually determined masks by experts were used as ground truth to assess the performance of the deep learning model.

**Results:**

The deep learning model achieved a mean Dice score of 0.926 ± 0.034 (0.921 ± 0.026 for the left testis and 0.926 ± 0.034 for the right testis) in the validation cohort and a mean Dice score of 0.922 ± 0.02 (0.931 ± 0.019 for the left testis and 0.932 ± 0.022 for the right testis) in the testing cohort. There was strong correlation between the manual and automated TV (*R*^2^ ranging from 0.974 to 0.987 in the validation cohort; R^2^ ranging from 0.936 to 0.973 in the testing cohort). The volume differences between the manual and automated measurements were 0.838 ± 0.991 (0.209 ± 0.665 for LTV and 0.630 ± 0.728 for RTV) in the validation cohort and 0.815 ± 0.824 (0.303 ± 0.664 for LTV and 0.511 ± 0.444 for RTV) in the testing cohort. Additionally, the deep-learning model exhibited excellent reproducibility (intraclass correlation >0.9) in determining TV.

**Conclusion:**

The MRI-based deep learning model is an accurate and reliable tool for measuring TV.

## Introduction

The testis is an important organ for male spermatogenesis and testosterone synthesis (1–3). As the seminiferous tubules account for approximately 80–90% of the testicular mass, the testicular volume (TV) reflects sperm and hormonal status (1, 2, 4–7). In clinical practice, the TV is an essential parameter for monitoring testicular functions and pathologies (2). An increased TV is the earliest sign of pubertal gonadotropin elevation; thus, TV measurements are used to monitor testicular development and pubertal status. Normal spermatogenesis occurs only when the total TV is normal or approximately normal, and the amount of TV loss is associated with the degree of spermatogenesis disorder (5). The TV has been proven to be related to semen profiles, and TV measurements are key components of male infertility evaluations (1, 6, 8). Therefore, accurate and individualized TV measurements may improve the diagnosis and treatment of patients with various disorders that affect testicular growth and fertility (2, 4, 7).

Several methods are used to assess TV, including calipers, different types of orchidometers, and ultrasonography (US) (4–6, 8–13). Clinical methods, such as calipers and orchidometers, are subjective in nature and tend to overestimate the true TV due to potential interference from the adjacent soft tissue, such as the epididymis, scrotal skin, and subcutaneous tissues, particularly in the case of small testes and hydrocele (5, 6, 11–14). Formula-derived US is generally used as the standard method for determining TV nowadays. TV is usually calculated as length (L) × width (W) × height (H) × constant (C), where C is a correction factor (often recommended as 0.71 or 0.52), and the length, width, and height are the sizes of the testicular axes determined by the sonographers (2, 15). However, the formula-derived measurement is recognized as a rough estimate of the TV, because the testis is an elastic and compressible organ with a shape that is neither uniform nor necessarily ellipsoid (5, 11). TV measurements obtained *via* US have been proven to vary from study to study, formula to formula, and examiner to examiner (2, 9, 10, 15); thus, establishing normative TV values and cutoffs for distinguishing pathological conditions has proven challenging, limiting the standardized use of TV in clinical practice (2, 5, 11, 15). Therefore, efforts are still needed to develop methods that are accurate, convenient, and individualized.

Recently, deep learning models have demonstrated great potential in attaining highly accurate volume measurements (16). These models first automatically segment the targets using deep learning algorithms, and then calculate the volumes of the targets by multiplying the voxel size by the voxel number (17). Measurement accuracy relies more on the precision of automatic segmentation results than on the match between the shape of the targets and the formula employed (18). Highly accurate auto-segmentation and volume estimation using deep learning models have been reported in several organs and pathologies, such as brain tumors, the liver, the kidney, the spleen, and the inner ear (16–23). However, the performance of deep learning models in estimating testicular volume has not been reported previously.

Therefore, in this study, a deep learning model, specifically, a ResUNet algorithm, is used to automatically segment the testes on T2-weighted imaging (T2WI) and calculate testicular volume. Masks manually defined by experts served as the reference standard for evaluating the performance of the deep learning model. A subset of subjects was scanned multiple times to evaluate the repeatability of the segmentation results. Our findings demonstrate that the T2WI-based deep learning model is an accurate and reliable tool for TV measurement.

## Materials and methods

Our institutional review board approved this study, and the requirement for informed consent was waived.

### Study population

The study population consisted of a retrospective dataset and a prospective dataset. For the collection of the retrospective data, we searched the electronic database of our institution from February 2014 to September 2021 for males who underwent magnetic resonance imaging (MRI) of the scrotum for any reason, such as scrotal pain and infertility. The inclusion criteria were defined as follows: (1) Both testes exhibited anatomically intact morphology, (2) no visible intratesticular lesions were present, and (3) patients underwent 3.0 T MRI scans of the scrotum, with available T2WI included in the MRI protocol. The exclusion criteria were defined as follows: (1) undescended testes, (2) testis was too small to observe in three image slices, (3) the quality of the MR images was poor, (4) patients underwent treatment, such as orchiectomy, partial orchiectomy, testis-sparing surgery, radiotherapy, or chemotherapy, due to testicular diseases, and (5) patients underwent androgen deprivation therapy due to prostate cancer. Finally, a total of 200 consecutive patients (400 testes) were enrolled in the retrospective dataset. This dataset was divided into training and independent validation cohorts according to a random distribution of 8:2. The training cohort was employed to train the network, while the validation cohort was used to evaluate the segmentation performance of the network.

A prospectively collected dataset comprising of ten healthy volunteers was used as the testing cohort. Each volunteer was scanned 5 times. The subjects were repositioned (removed from the scanner and asked to sit up and move on the bed) and reregistered on the scanner console between scans in each session; thus, all scans were treated as separate measurements. In addition, we attempted to vary the acquisition geometry between each scan while still acquiring full testes coverage. The testing data were used to assess the reproducibility of the MRI-based measurements.

### MRI acquisition

All images were acquired using a 3 T MAGNETOM Skyra (Siemens Healthcare, Erlangen, Germany) and an anterior 18-element body matrix coil combined with a posterior 32-channel spine coil. Multiple sequences were scanned, but only the T2-weighted turbo spin–echo sequences were used in this study. The transverse T2WI were acquired using the following parameters: 3 mm slice thickness, 0 mm slice gap, 6,500 ms repetition time, 104 ms echo time, 180 × 180 in field of view and 384 × 320 acquisition matrix.

Notably, the T2WI parameters were consistent with the standardized technical requirements for scrotal imaging recommended by the Scrotal and Penile Imaging Working Group of the European Society of Urogenital Radiology (24, 25). The acquisition time of the transverse T2WI was approximately 180 s.

### Manual segmentation

The manual segmentation results were used as the ground truth. Manual segmentation was performed using ITK SNAP software (version 3.4.0; www.itksnap.org). Three-dimensional binary masks of the entire testes were generated by tracing the testicular boundaries slice-by-slice on the transverse T2WI. The non-testicular parenchyma area, including the epididymis and mediastinum, was excluded from the manual segmentation. Manual segmentation was carried out by two radiologists (observer 1, with 10 years of experience in interpreting MRI of scrotum, and observer 2, with 5 years of experience in interpreting MRI of scrotum) in a blind manner. For the manual segmentation of the retrospective dataset, the images were collectively analyzed by the two observers, and discrepancies were resolved through discussion until a consensus was reached.

For the initial segmentation of the prospective dataset, which served as the ground truth, readers 1 and 2 collectively segmented the images [region of interest (ROI) A] for all 5 repeated acquisitions. Then, 1 month after the initial segmentation, readers 1 (ROI B) and 2 (ROI C) independently segmented all 5 repeated acquisitions to assess the inter- and intra-observer variability of the manual segmentation. The volume of each testis was computed by multiplying the voxel volume by the number of voxels in each testis mask. Subsequently, the total testicular volume (TTV) was calculated by summing the volumes of both testes.

### Automated segmentation using ResUNet

All images were preprocessed, including resampling, normalization, cropping, and padding, to generate homogeneous MRI volumes. First, all volumes were resampled to the same voxel size of 0.46875 mm × 0.46875 mm × 1 mm. Subsequently, the intensities of each volume were normalized to the range [−1, 1]. The architecture of the model is based on the ResUNet algorithm (7, 26–28). Briefly, the model has encoding, bridge, and decoding parts. The encoding part encodes the input image into compact representations, while the decoding part recovers the representations for pixel-wise categorization. The bridge part connects the encoding and decoding paths. The ResUNet algorithm was implemented in Python 3.9.7 using PyTorch version 1.8.0. The network uses a Tversky loss function. The model was trained with a batch size of 1 over 200 epochs using the Adam optimizer. We set the initial learning rate to 0.0001 and trained the network for 600 iterations, reducing the learning rate to 80% of the current value every 20 iterations. The ResUNet model was trained using RTX 2080Ti GPUs (NVIDIA).

### Statistical analysis

The baseline demographics are reported in the form of mean ± standard deviation (SD). The accuracy of the deep learning model was assessed by comparing the automated segmentation results with the manual segmentation results. The reliability of the manual segmentation results and the reproducibility of the deep learning model were evaluated using the testing dataset. Voxel-based similarity metrics (e.g., Dice score) and surface-based similarity metrics (e.g., Hausdorff distance) were employed to evaluate the overlap between masks. In addition, volume differences, including actual volume difference and percentage volume difference, were computed. The mean coefficient of variation (CoV; defined as SD/mean) and the intraclass correlation coefficient (ICC) were used to assess repeatability. Bland–Altman and regression analyses were conducted to evaluate the correlation between manual TV and automated TV.

## Results

### Patients

**Figure 1:**
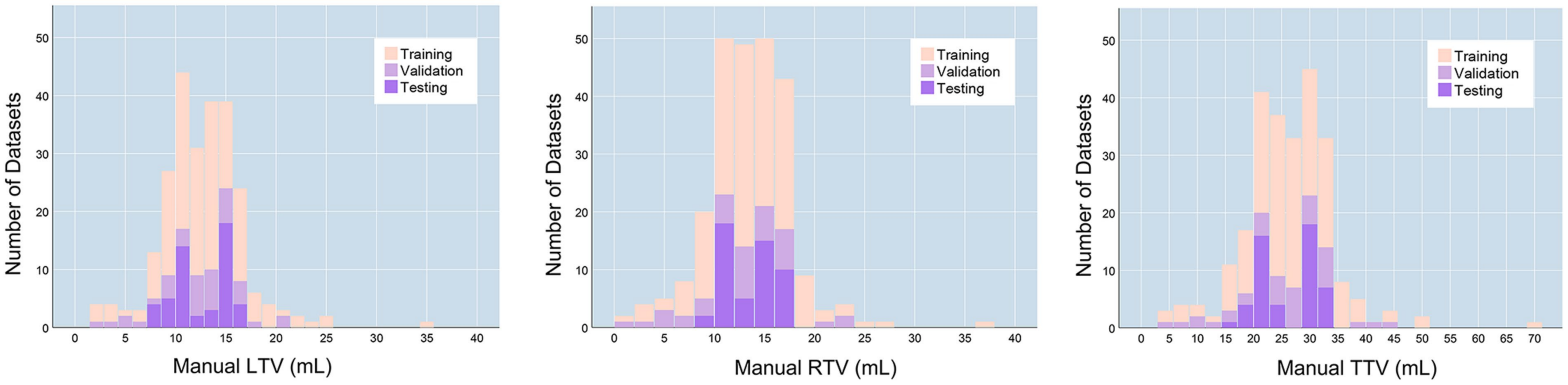
TV distributions. LTV, left testicular volume; RTV, right testicular volume; TTV, total testicular volume.

The final training dataset included MRI scans of 160 cases from 160 patients (aged 9–74 years; mean age 34.713 ± 14.542 years). In the training cohort, the average left testicular volume (LTV) was 12.539 ± 2.625 mL (1.471–34.628 mL), the average right testicular volume (RTV) was 13.579 ± 4.366 mL (1.824–36.601 mL), and the average total testicular volume (TTV) was 26.333 ± 8.357 mL (3.295–71.229 mL). The validation dataset included MRI scans of 40 cases from 40 patients (aged 11–70 years; mean age 33.4 ± 13.388 years). In the validation dataset, the average LTV was 12.351 ± 4.133 mL (2.356–21.373 mL), the average RTV was 12.672 ± 4.821 mL (1.539–23.126 mL), and the average TTV was 25.023 ± 8.676 mL (4.629–43.276 mL). The prospective testing dataset included MRI scans of 50 cases from 10 healthy volunteers (aged 13–30 years; mean age 19.7 ± 5.33 years). In the testing dataset, the average LTV was 12.539 ± 2.625 mL (8.162–16.072 mL), the average RTV was 13.549 ± 2.505 mL (8.187–16.945 mL), and the average TTV was 26.089 ± 5.052 mL (16.833–32.354 mL). The characteristics of the enrolled patients are provided in Table 1. The distributions of the TV in the training, validation and testing datasets are shown in Figure 1.

**Table 1:** Characteristics of the enrolled patients.

Patients	Number of patients	Number of datasets	Mean Age (years)	LTV (mL)	RTV (mL)	TTV (mL)
Training Cohort	160	160	34.713 ± 14.542	12.753 ± 4.342	13.579 ± 4.366	26.333 ± 8.357
Validation Cohort	40	40	33.400 ± 13.388	12.351 ± 4.133	12.672 ± 4.821	25.023 ± 8.676
Testing Cohort	10	50	19.700 ± 5.330	12.539 ± 2.625	13.549 ± 2.505	26.089 ± 5.052

LTV, left testicular volume; RTV, right testicular volume; TTV, total testicular volume.

All values are quoted as mean ± SD.

### Reliability of the manual segmentation results

The healthy volunteers in the testing dataset were utilized to analyze the reproducibility of the manual segmentation results, as healthy testes have more consistent morphologies and therefore provide better performance for repeatability evaluation. First, based on masks manually determined by different experts, interobserver variability of the manual segmentation results was evaluated, as shown in Supplementary Table S1. The overlap between different manual masks was analyzed using similarity metrics, including the Dice score, Jaccard index, and Hausdorff distance. The actual volume difference was calculated. Next, based on the 5 repeated scans in the testing dataset, the intra-observer variability of the manual segmentation results was assessed. As shown in Table 2, the intra-observer repeatability of the manual TV was excellent (ICC > 0.9), regardless of the experiments of the observers or whether the manual segmentations were performed independently by one radiologist or collectively by two radiologists.

**Table 2:** Intra-observer repeatability of the manual measurements.

Observer	Testis	CoV (%)	ICC
Intra ROI A	Left	2.931 ± 1.291	0.971
	Right	3.829 ± 2.792	0.946
	Total	2.487 ± 1.193	0.981
Intra ROI B	Left	2.685 ± 0.965	0.977
	Right	3.991 ± 2.192	0.949
	Total	2.842 ± 0.813	0.978
Intra ROI C	Left	2.797 ± 0.842	0.976
	Right	4.051 ± 2.833	0.946
	Total	2.753 ± 1.420	0.979

CoV, coefficient of variation.

All CoV values are represented as the mean ± SD. ROI A was segmented collectively by readers 1 and 2. ROI B was independently segmented by reader 1, and ROI C was independently segmented by reader 2.

### Accuracy of the deep learning model

**Figure 2:**
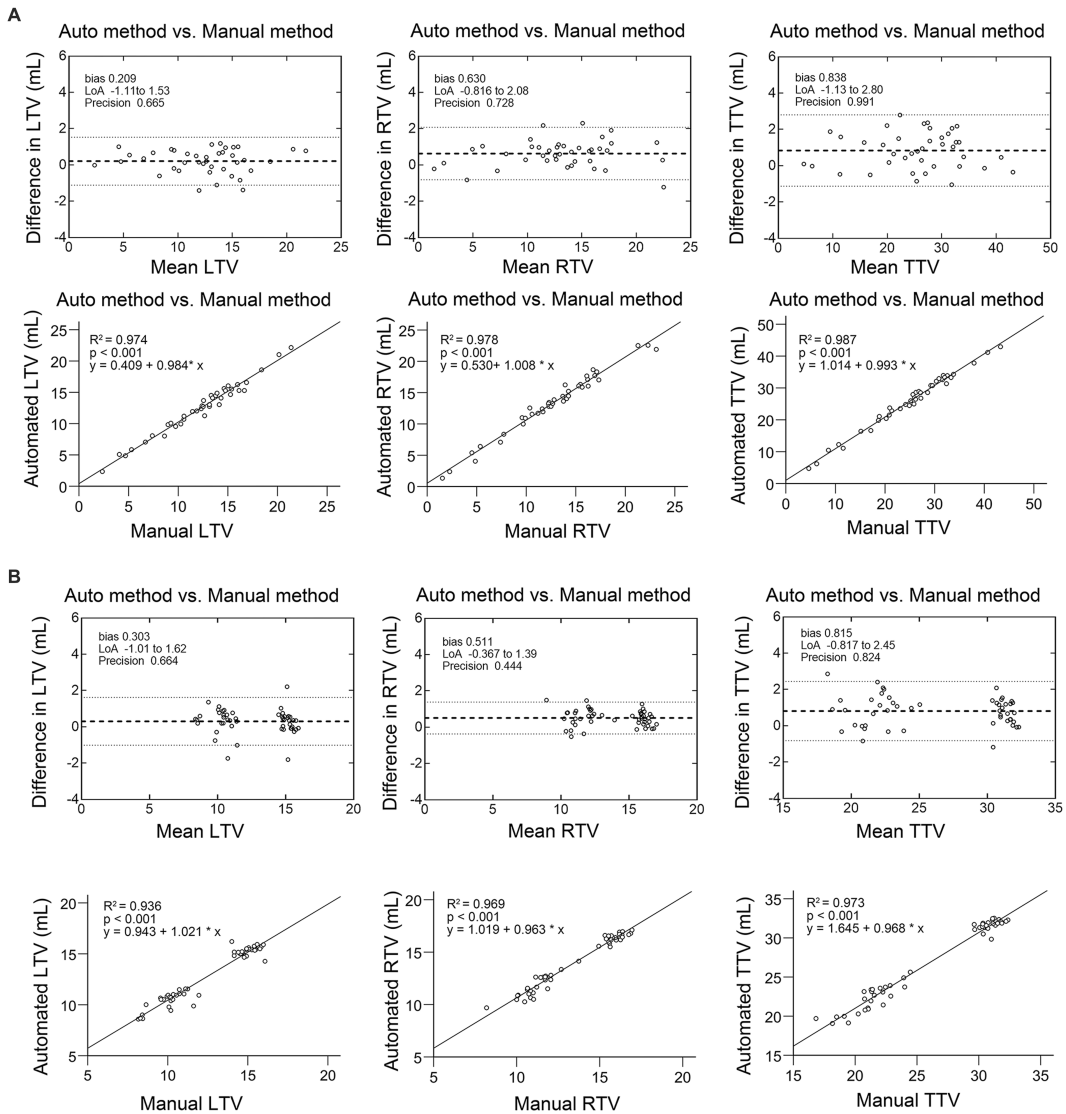
Scatter plot and Bland–Altman graph showing the difference between automated TV and manual TV. **(A)** Validation dataset. **(B)** Testing dataset. In the Bland–Altman graph, the solid lines show the actual mean difference (bias), and the dotted lines show 95% limits of agreements (LoAs).

**Figure 3:**
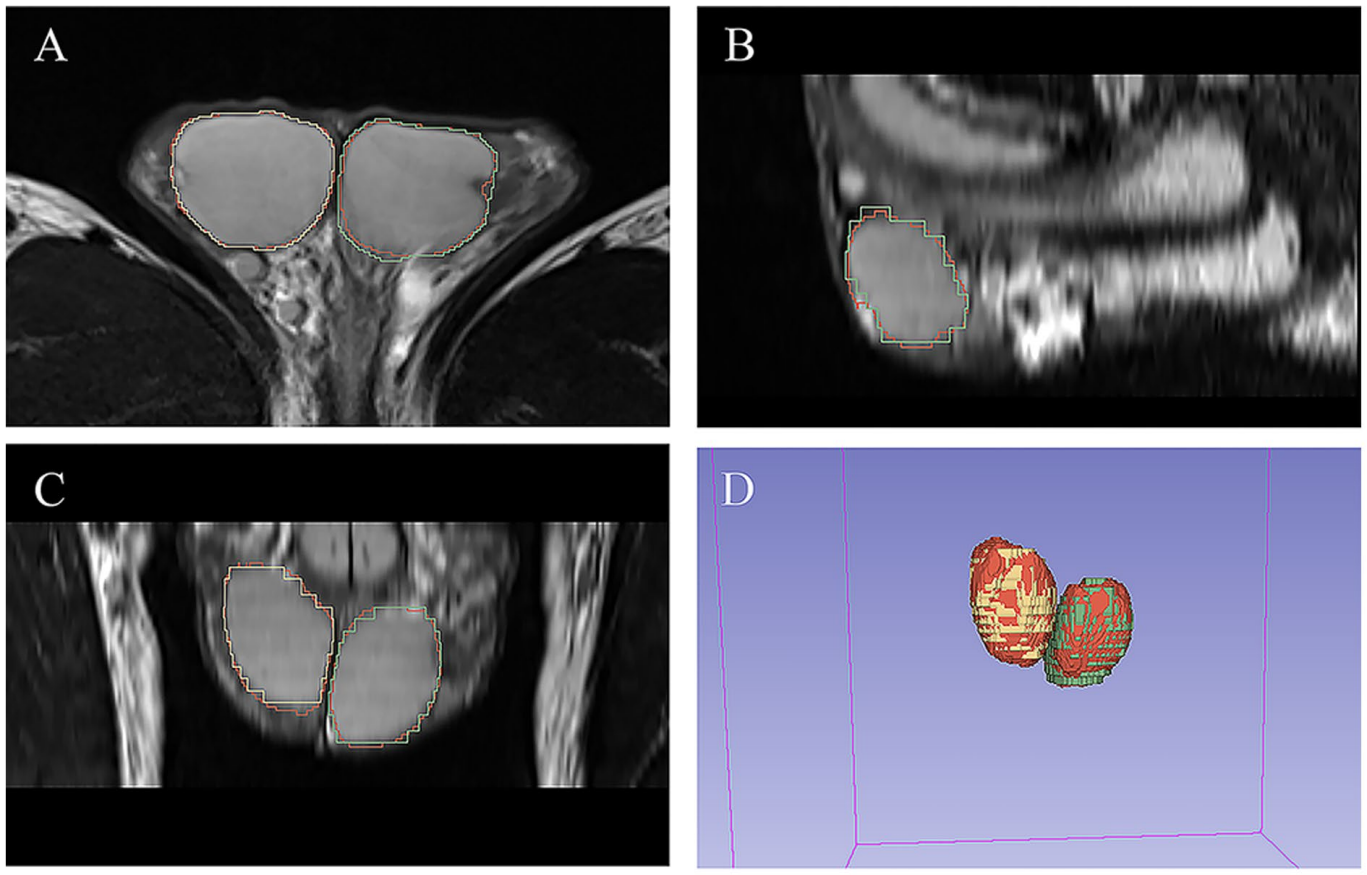
Example of manual and automated segmentation of the testes. **(A)** Axial view. **(B)** sagittal view. **(C)** Coronal view. **(D)** 3D volume. The manual mask generated by experts for left testis is shown in blue, the manual mask for right testis is shown in green, and the automatically generated mask is shown in red.

As shown in Table 3, there was excellent similarity between the automatic and manual segmentations, with a mean Dice score of 0.922 ± 0.02 (0.921 ± 0.026 for the left testis and 0.926 ± 0.034 for the right testis) in the validation cohort and a mean Dice score of 0.931 ± 0.018 (0.931 ± 0.019 for the left testis and 0.932 ± 0.022 for the right testis) in the testing cohort. Linear regression analysis indicated a strong positive correlation (R2 ranging from 0.974 to 0.987, *p* < 0.001 for the validation cohort; R2 ranging from 0.936 to 0.973, *p* < 0.001 for the testing cohort) between the manual TV and automated TV (Figure 2, Supplementary Figure S1). For TTV, the bias (mean) and precision (SD) of the automated measurements were 0.838 and 0.991 in the validation cohort and 0.815 and 0.824 in the testing cohort. For LTV, the bias and precision of the automated measurements were 0.209 and 0.665 in the validation cohort and 0.303 and 0.664 in the testing cohort. For RTV, the bias and precision of the automated measurements were 0.630 and 0.728 in the validation cohort and 0.511 and 0.824 in the testing cohort. In terms of volume error, the actual volume differences between manual measurements and automated measurements were 0.209 ± 0.665 for LTV, 0.630 ± 0.728 for RTV, and 0.838 ± 0.991 for TTV in the validation cohort. In the testing cohort, the percentage volume differences between manual measurements and automated measurements were 0.303 ± 0.664 for LTV, 0.511 ± 0.444 for RTV, and 0.815 ± 0.824 for TTV. The percentage volume differences between manual measurements and automated measurements were 2.192 ± 6.129% for LTV, 4.654 ± 7.355% for RTV, and 3.711 ± 4.983% for TTV in the validation cohort. In the testing cohort, the percentage volume differences between manual measurements and automated measurements were 2.621 ± 5.580% for LTV, 3.909 ± 3.856% for RTV, and 3.266 ± 3.668% for TTV. Figure 3 illustrates an example of manual segmentation alongside the corresponding automated segmentation generated by the deep learning model.

**Table 3:** The accuracy of the deep learning model.

Datasets	testis	Dice score	Jaccard index	Hausdorff distance (95th percentage)	Actual volume difference (mL)	Percentage volume difference (%)
Training	Left	0.918 ± 0.044	0.852 ± 0.064	1.412 ± 0.756	0.388 ± 0.761	2.639 ± 7.475
	Right	0.926 ± 0.034	0.864 ± 0.051	1.886 ± 7.471	0.578 ± 0.816	4.337 ± 8.170
	Total	0.923 ± 0.029	0.859 ± 0.046	1.926 ± 7.272	0.967 ± 1.231	3.627 ± 6.153
Validation	Left	0.921 ± 0.026	0.854 ± 0.043	1.364 ± 0.687	0.209 ± 0.665	2.192 ± 6.129
	Right	0.921 ± 0.027	0.856 ± 0.046	1.389 ± 0.747	0.630 ± 0.728	4.654 ± 7.355
	Total	0.922 ± 0.02	0.856 ± 0.033	1.386 ± 0.482	0.838 ± 0.991	3.711 ± 4.983
Testing	Left	0.931 ± 0.019	0.871 ± 0.033	1.182 ± 0.426	0.303 ± 0.664	2.621 ± 5.580
	Right	0.932 ± 0.022	0.873 ± 0.037	1.222 ± 0.515	0.511 ± 0.444	3.909 ± 3.856
	Total	0.931 ± 0.018	0.872 ± 0.030	1.183 ± 0.424	0.815 ± 0.824	3.266 ± 3.668

All values are represented as the mean ± SD.

### Repeatability of the deep learning model

Based on the 5 repeated scans in the testing dataset, the repeatability of the MR-based automated measurements was evaluated (Table 4). Across the 5 different measurements, the automated method demonstrated excellent repeatability, with ICCs of 0.973 for LTV, 0.970 for RTV, and 0.982 for TTV. The mean CoV across the 5 different measurements were 2.964% ± 1.873% for LTV, 2.556% ± 1.690% for RTV, and 2.156% ± 1.352% for TTV, which were similar to the CoV of the manual methods (*p* = 0.961, *p* = 0.118, and *p* = 0.343, respectively).

**Table 4:** Comparison of the repeatability between the manual and automated measurements.

Testis	ICC		CoV (%)		
	Manual	Auto	Manual	Auto	*p* ^*^
Left	0.971	0.973	2.931 ± 1.291	2.964 ± 1.873	0.961
Right	0.946	0.967	3.829 ± 2.792	2.779 ± 1.853	0.118
Total	0.981	0.984	2.487 ± 1.193	2.047 ± 1.319	0.343

CoV, coefficient of variation.

All CoV values are represented as the mean ± SD.

*Paired student *t* test.

## Discussion

In this study, utilizing retrospectively collected patient data and prospectively collected data from healthy volunteers, we developed a deep learning model to automatically segment the testes and measure TV. The deep learning model achieved accurate segmentation and provided reliable TV measurements. For the first time, we report that the MR-based deep learning model holds promise as a valuable tool for TV measurements.

As an essential parameter for monitoring testicular functions and pathologies, TV measurements have long been a subject of research focus (1, 2, 29). Over the past decades, efforts have been made to improve the accuracy of TV measurements, and formula-derived US measurements are generally used as the standard method for TV determination (1, 6, 10). However, the testis is an elastic and compressible organ whose elasticity varies across different developmental stages and pathological conditions. Moreover, the testis does not always conform to a strictly ellipsoidal shape. Consequently, precise and individualized measurements cannot be achieved through formula-derived approaches (5, 15). Recently, deep learning models have been reported to obtain highly accurate volume measurements of various organs and tissues, including the lungs, liver, kidney, spleen, and brain tumors (16, 18–21). For example, Daniel AJ et al. enrolled 30 healthy volunteers and 30 chronic disease patients, reporting that their deep learning model allowed for accurate segmentation and volume measurements of the kidney, yielding a mean Dice score of 0.93 ± 0.01 and a mean volume difference of 1.2 ± 16.2 mL (20). Modanwal G et al. demonstrated that a deep learning model enabled accurate segmentation of the liver and spleen in non-contrast computed tomography images, achieving a Dice coefficient of 0.95 in an independent validation cohort (16). In this study, utilizing retrospectively collected patient data (*N* = 200, comprising the training and independent validation cohorts) and prospective data from healthy volunteers (*N* = 50, serving as the testing cohort), we found that the ResUNet deep learning model enabled accurate TV measurements. This was reflected in mean Dice scores of 0.926 ± 0.034 and 0.922 ± 0.02, respectively in the validation and testing cohorts, along with volume differences of 0.838 ± 0.991 and 0.815 ± 0.824, respectively in the validation and testing cohorts. The possible reason might be as follows. On one hand, the testis exhibits relatively uniform characteristics in T2-weighted MR images, and the ResUNet model has previously demonstrated exceptional performance in automatically segmenting organs and tissues with repetitive structures (22–24, 26–28, 30). On the other hand, MRI, especially T2WI, provides excellent soft tissue contrast, facilitating the clear delineation of the tunica albuginea and tunica vaginalis that enclose the testes. Consequently, the testes can be accurately differentiated from the surrounding tissue in T2WI.

Another point of concern in automated volume measurement is its repeatability. Longitudinal follow-up of TV may be necessary in certain clinical settings, such as closely monitoring changes in pubertal status, tracking testicular involvement in pathological processes, and assessing the impact of chemotherapeutic or hormonal agents on the testes. TV measurements must exhibit high reproducibility to be valuable in longitudinal studies (4, 5, 31). In this study, we obtained 5 scans for each volunteer in the testing cohort to investigate the reproducibility of the MR-based measurement. Our results showed that MR-based deep learning model have small variations and excellent reproducibility; Thus is a reliable tool for TV measurements. In addition, our results also suggest that the MR-based manual measurements showed excellent inter- and intra-observer repeatability, regardless of the experiments of the observer or whether the manual segmentations were performed independently by one radiologist or collectively by two radiologists. These results demonstrate the reliability and rationality of the proposed MR-based measurement approach. One possible reason for these findings is that the testes could be well discriminated from the surrounding tissue in the T2WI.

Although MRI provides richer morphological and functional information and is less dependent on operator experience, US remains the first choice for diagnostic imaging of the scrotum (15, 24, 29). MRI is recommended as a valuable alternative diagnostic tool for investigating scrotal pathology (24, 25). The main reason is that US is faster, more easily accessible, and more convenient, whereas multiplane and multimodal imaging are needed for scrotal MRI (24). However, in this study, the deep learning model was trained on only transverse T2WI, which takes only about 180 s to obtain the images. Therefore, the MRI-based deep-learning model proposed in this study is low time consuming, reliable and individualized.

This study has several limitations. First, there is a lack of data on US-derived measurements to conduct a comparison between US-derived measurements and MRI-based measurements. Second, the retrospective data served as training and validation cohorts containing heterogeneous patient populations, including infertility, hydrocele, scrotal pain, etc. A deep learning model trained with heterogeneous patient data can be clinically significant since the TV is typically used to assess patients with a variety of disorders that may affect testicular growth and fertility, such as infertility and varicocele. Third, this work was a single-center study. Multicenter studies are needed to validate our findings. Notably, the scan parameters used in this study were consistent with the standardized scrotal MRI technical requirements recommended by the Scrotal and Penile Imaging Working Group of the European Society of Urogenital Radiology, suggesting the universality of the proposed deep learning model.

## Conclusion

In conclusion, the proposed MRI-based deep learning model is an accurate and reliable tool for the segmentation and volume measurement of the testes.

## Data availability statement

The raw data supporting the conclusions of this article will be made available by the authors, without undue reservation.

## Ethics statement

The studies involving humans were approved by the Ethical Committee of Tongji Hospital of Tongji Medical College of Huazhong University of Science and Technology. The studies were conducted in accordance with the local legislation and institutional requirements. Written informed consent for participation was not required from the participants or the participants’ legal guardians/next of kin in accordance with the national legislation and institutional requirements.

## Author contributions

KS: Conceptualization, Methodology, Project administration, Writing – original draft, Writing – review & editing. CF: Conceptualization, Methodology, Project administration, Writing – original draft, Writing – review & editing. ZF: Conceptualization, Methodology, Project administration, Writing – review & editing. XM: Data curation, Methodology, Software, Writing – review & editing. YW: Formal analysis, Methodology, Software, Writing – review & editing. ZS: Data curation, Formal analysis, Writing – review & editing. YL: Data curation, Formal analysis, Writing – original draft. WC: Data curation, Formal analysis, Writing – review & editing. XY: Data curation, Formal analysis, Writing – review & editing. PZ: Data curation, Formal analysis, Writing – review & editing. QL: Data curation, Formal analysis, Writing – review & editing. LX: Conceptualization, Funding acquisition, Project administration, Supervision, Writing – review & editing.

## Supplementary material

Supplementary Figure S1 Scatter plot and Bland–Altman graph showing the difference between automated TV and manual TV in the training dataset. In the Bland–Altman graph, solid lines represent the actual mean difference (bias), while dotted lines represent the 95% limits of agreement (LoAs).
