# Supplementary figures and images for: Magnetic resonance imaging based deep-learning model: a rapid, high-performance, automated tool for testicular volume measurements
Source: Front Med (Lausanne). 2023 Sep 19;10:1277535. doi: 10.3389/fmed.2023.1277535 (PMC10546058; doi:10.3389/fmed.2023.1277535)

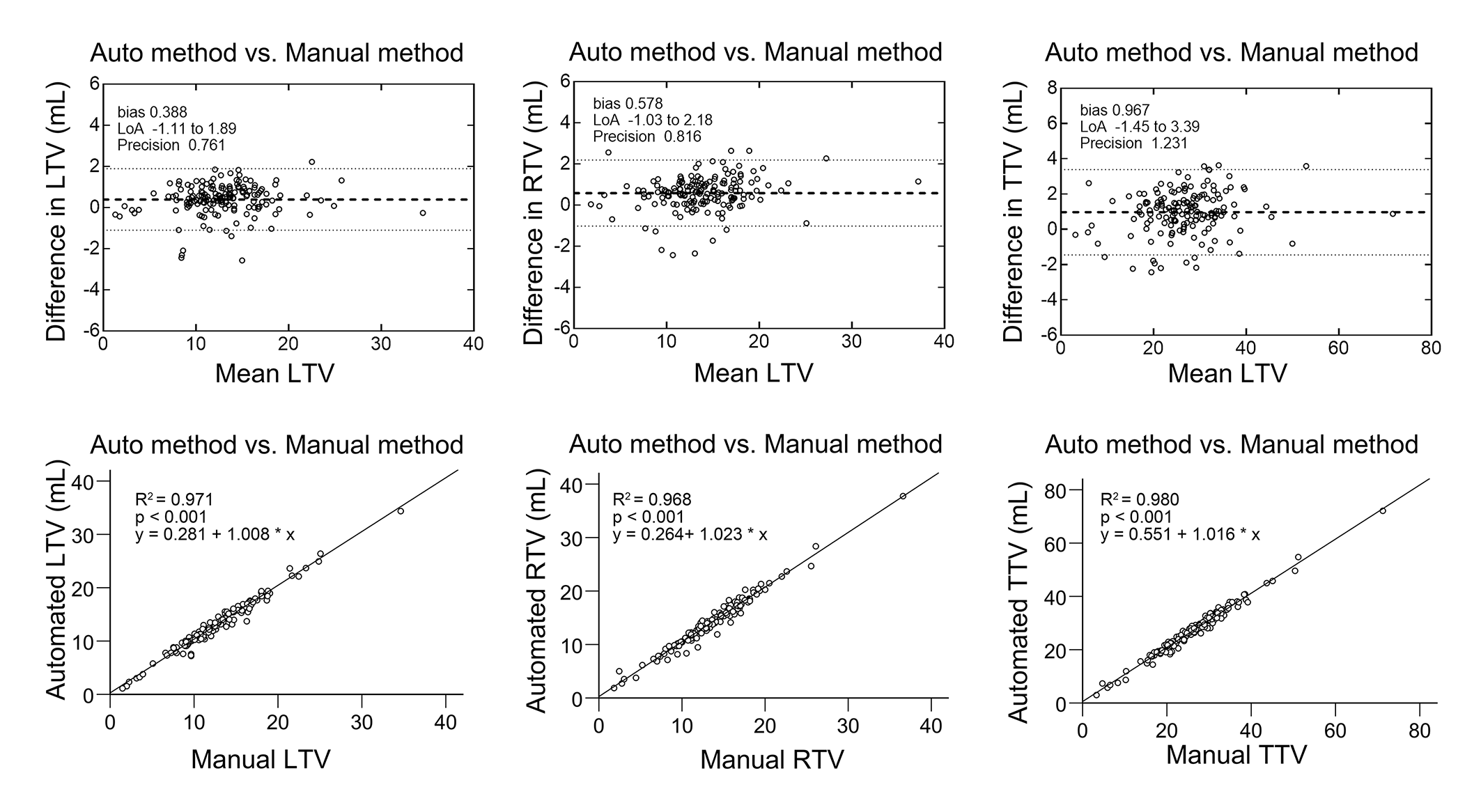

Supplement: Supplementary Figure S1 — Scatter plot and Bland–Altman graph showing the difference between automated TV and manual TV in the training dataset. In the Bland–Altman graph, solid lines represent the actual mean difference (bias), while dotted lines represent the 95% limits of agreement (LoAs). [file Image_1.TIF]
