# Supplementary material for: Magnetic resonance imaging based deep-learning model: a rapid, high-performance, automated tool for testicular volume measurements
Source: Front Med (Lausanne). 2023 Sep 19;10:1277535. doi: 10.3389/fmed.2023.1277535 (PMC10546058; doi:10.3389/fmed.2023.1277535)
Supplement: Supplementary file 2 [file Table_1.DOCX]

Table S1. Metrics comparing each combination of manual masks.

|  | testis | Dice score | Jaccard index (mm) | Hausdorff distance (mm) (95^th^ percentage) | Actual volume difference (mL) |
| --- | --- | --- | --- | --- | --- |
| ROI A vs. ROI B | Left | 0.971 ± 0.009 | 0.945 ± 0.016 | 0.858 ± 0.305 | 0.137 ± 0.345 |
|  | Right | 0.973 ± 0.012 | 0.947 ± 0.023 | 0.818 ± 0.322 | 0.171 ± 0.318 |
|  | Total | 0.972 ± 0.008 | 0.946 ± 0.015 | 0.821 ± 0.297 | 0.308 ± 0.533 |
| ROI A vs. ROI C | Left | 0.968 ± 0.011 | 0.938 ± 0.020 | 0.958 ± 0.290 | 0.347 ± 0.330 |
|  | Right | 0.970 ± 0.013 | 0.942 ± 0.024 | 0.873 ± 0.360 | 0.372 ± 0.284 |
|  | Total | 0.969 ± 0.010 | 0.940 ± 0.019 | 0.908 ± 0.335 | 0.719 ± 0.500 |
| ROI B vs. ROI C | Left | 0.972 ± 0.009 | 0.947 ± 0.016 | 0.783 ± 0.318 | 0.210 ± 0.295 |
|  | Right | 0.974 ± 0.009 | 0.949 ± 0.016 | 0.806 ± 0.328 | 0.201 ± 0.268 |
|  | Total | 0.973 ± 0.006 | 0.948 ± 0.012 | 0.783 ± 0.267 | 0.411 ± 0.429 |

All values are represented as the mean ± SD.

ROI A was segmented collectively by readers 1 and 2. ROI B was independently segmented by reader 1, and ROI C was independently segmented by reader 2.
